# Family-based association study of *ZNF804A* polymorphisms and autism in a Han Chinese population

**DOI:** 10.1186/s12888-019-2144-1

**Published:** 2019-05-23

**Authors:** Ziqi Wang, Tian Zhang, Jing Liu, Han Wang, Tianlan Lu, Meixiang Jia, Dai Zhang, Lifang Wang, Jun Li

**Affiliations:** 1 Peking University Sixth Hospital, No. 51, Hua Yuan Bei Road, Beijing, 100191 China; 2 Peking University Institute of Mental Health, Beijing, 100191 China; 3 NHC Key Laboratory of Mental Health (Peking University), Beijing, 100191 China; 4 National Clinical Research Center for Mental Disorders (Peking University Sixth Hospital), Beijing, 100191 China; 5 Peking-Tsinghua Center for Life Sciences, Peking University, Beijing, 100871 China; 6 PKU-IDG/McGovern Institute for Brain Research, Peking University, Beijing, 100871 China

**Keywords:** Autism, *ZNF804A*, Single-nucleotide polymorphism, Family-based association study

## Abstract

**Background:**

Autism is a complex neurodevelopmental disorder with high heritability. Zinc finger protein 804A (*ZNF804A*) was suggested to play important roles in neurodevelopment. Previous studies indicated that single-nucleotide polymorphism (SNP) rs1344706 in *ZNF804A* was strongly associated with schizophrenia and might influence social interaction. Only one study explored the significance of *ZNF804A* polymorphisms in autism, which discovered that rs7603001 was nominally associated with autism. Moreover, no previous study investigated the association between *ZNF804A* and autism in a Han Chinese population. Here, we investigated whether these two SNPs (rs1344706 and rs7603001) in *ZNF804A* contribute to the risk of autism in a Han Chinese population.

**Methods:**

We performed a family-based association study in 640 Han Chinese autism trios. Sanger sequencing was used for sample genotyping. Then, single marker association analyses were conducted using the family-based association test (FBAT) program.

**Results:**

No significant association was found between the two SNPs (rs1344706 and rs7603001) in *ZNF804A* and autism (*P* > 0.05).

**Conclusions:**

Our findings suggested that rs1344706 and rs7603001 in *ZNF804A* might not be associated with autism in a Han Chinese population.

**Electronic supplementary material:**

The online version of this article (10.1186/s12888-019-2144-1) contains supplementary material, which is available to authorized users.

## Background

Autism is a complex neurodevelopmental disorder, characterized by early-onset impairments in social interaction and communication, repetitive behaviors, and restricted interests. The worldwide prevalence of autism was estimated to about 1% with a male-to-female ratio of 3–4:1 [1, 2]. The high concordance between identical twins (nearly 90%) indicated the critical role of genetic factors in the pathogenesis of autism [3, 4]. Despite the research progress made over the past few decades, the genetic pathogenesis of autism remains largely unclear [5–8].

Zinc finger protein 804A (*ZNF804A*) is located in 2q32.1 and encodes a protein containing a C2H2-type zinc finger domain, which participates in DNA binding and transcription regulation [9–11]. *ZNF804A* is highly expressed in the human brain with a subcellular distribution in somatodendritic compartments [12]. Besides, high expression of *Znf804a* was found in developing brains of mice, especially during the late developmental stages (> 20 weeks) [13]. Furthermore, functional studies suggested that *ZNF804A* might affect the expression of the genes involved in neurite outgrowth, synapse formation, and dopaminergic transmission [11, 14]. Postmortem studies of autistic individuals detected reduced expression of *ZNF804A* in the anterior cingulate gyrus (ACG), which was implicated in social behaviors and cognition [15, 16]. Altogether, these findings suggested that *ZNF804A* might play important roles in neurodevelopment.

Previous studies indicated that single-nucleotide polymorphism (SNP) rs1344706 in *ZNF804A* was strongly associated with schizophrenia (SCZ) [13, 17–21]. This disorder was suggested to overlap with autism partially in clinical phenotypes and susceptibility genes [22]. A neuroimaging study reported that the risk allele A of rs1344706 exerted a significant allele-dose effect in parts of the theory of mind (ToM) network, such as the left inferior prefrontal cortex, which was associated with social impairments in autism [23, 24]. ToM refers to the ability of understanding the feelings or thoughts of others, which is crucial for social interaction [25, 26]. Impairments of this ability represent a core deficit of autism [27–29]. In addition, rs7603001 in *ZNF804A* was nominally associated with autism (*P* = 0.018) in 841 autistic families from the Autism Genetic Resource Exchange (AGRE), most of whom were white [15].

Given the important roles of *ZNF804A* in neurodevelopment and the lack of studies exploring the association between *ZNF804A* polymorphisms and autism in the Han Chinese population, we conducted a family-based association study of two SNPs (rs1344706 and rs7603001) in *ZNF804A* with 640 autism trios of Han Chinese ancestry.

## Material and methods

### Participants

A total of 640 autistic nuclear trios (autistic children and their biological parents) were included in the study. All participants were of Han Chinese ancestry and recruited at Peking University Sixth Hospital, China. The median age of diagnosis for autistic children was 4.75 (range 3–16) years. The sex ratio (male:female) was approximately 7:1, including 563 male and 77 female children.

The autistic children were independently evaluated by two senior psychiatrists according to the Diagnostic and Statistical Manual of Mental Disorders, fourth edition criteria for autism. Additional criteria for patient inclusion were Autism Behavior Checklist score ≥ 53 and Childhood Autism Rating Scale ≥35 [30, 31]. Children diagnosed with Asperger syndrome, Rett syndrome, pervasive development disorder not otherwise specified, fragile X syndrome, tuberous sclerosis, a previously identified chromosomal abnormality, dysmorphic features, or any other neurological conditions were excluded from the present study. All the parents were evaluated through unstructured interviews by two psychiatrists to confirm that they were not affected with autism spectrum disorder (ASD). Any individuals with familial (inherited) diseases (such as congenital deaf-mutism, hemophilia, and familial adenomatous polyposis) or severe mental disorders (such as SCZ, schizoaffective disorder and bipolar disorder) were excluded in the study.

### SNPs selection

Two polymorphisms in *ZNF804A* (rs1344706 and rs7603001) were selected in the present study. The criteria for SNPs selection were as follows: (1) SNPs reported association with autism or ASD were selected; (2) risk variants of other mental disorders (such as SCZ, depression, and bipolar disorder) were also taken into account; (3) The minor allele frequencies (MAF) of selected SNPs should be greater than 0.05 in the Han Chinese in Beijing, China (CHB). The genotyping data of SNPs were downloaded from the databases Ensembl GRCh37 Release 93 (http://grch37.ensembl.org/index.html) and dbSNP in National Center for Biotechnology Information (NCBI) (https://www.ncbi.nlm.nih.gov/snp) [32].

### DNA extraction and genotyping

Peripheral blood samples were collected from all participants in the morning. Genomic DNA was extracted using the Qiagen QIAamp DNA Mini Kit (Qiagen, Hilden, Germany) following the manufacturer’s instructions. NanoDrop Spectrophotometer (Thermo Fisher Scientific, Waltham, MA, USA) was used to confirm that the concentrations of the extracted DNA were greater than 40 ng/uL.

Genotyping for rs1344706 and rs7603001 was performed by Sanger DNA sequencing. All primers for the polymerase chain reaction (PCR) were designed through the Primer-BLAST tool of the NCBI (https://www.ncbi.nlm.nih.gov/tools/primer-blast/) according to the sequence of the forward strands provided by the NCBI human reference genome GRCh38 (hg38). The primers used for rs1344706 were as follows: forward, 5′-ATTGGGACGAGGAGAAAA-3′; and reverse, 5′-GTCAAATAAGCCTGAGGAAT-3′. The primers for rs7603001 were the following: forward, 5′-TTCCAGAAAGCCATTCGTGTGTA-3′; and reverse, 5′-GAGCACCAGGAGAAACCAGT-3′. The PCR amplification for the Sanger sequencing was performed in a 15-μL mixture consisting of 1 μL of genomic DNA, 7.5 μL of 2× Easy Tag SuperMix (TransGen Biotech, Beijing, China), and 1.5 μL of each primers. The reaction was initiated with an initial denaturation at 95 °C for 5 min, followed by 38 cycles of denaturation at 95 °C for 30 s, annealing at 60 °C for rs1344706 and 64 °C for rs7603001 for 30 s, and extension at 72 °C for 42 s, followed by a final extension at 72 °C for 7 min. Then, Sanger sequencing of the amplified products was outsourced to BGI (Beijing, China). DNA sequencing was performed using the BigDye Terminator Cycle Sequencing Ready Reaction Kit with Ampli Taq DNA polymerase (PE Biosystem) and the ABI PRISM 377–96 DNA Sequencer (Applied Biosystem, Foster city, USA).

### Data analysis

Quanto, version 1.2.4 (http://biostats.usc.edu/software), was employed to evaluate the statistical power for risk allele detection [33]. The parameters were set to a population risk of 0.01, an estimated relative risk for common variants of 1.1 to 1.2, and a type I error rate of 0.05 (two-sided) under the log-additive model [15, 34]. The chi-square goodness-of-fit test was conducted to analyze the deviation from the Hardy–Weinberg equilibrium (HWE) for the genotype frequency distributions.

The family-based association test (FBAT) program, version 2.0.3, was used to check the Mendelian errors and reset the genotypes of the families with Mendelian errors to zero [35]. Then, single marker association analyses were conducted by the FBAT program under the additive and recessive inheritance models, respectively. All *P*-values calculated by the FBAT were two-sided. Bonferroni correction was applied to decrease the type I errors with a significance level of *P* < α/n (α = 0.05) [36]. The ratio of transmission to untransmission (T:U) for the alleles of each SNP was calculated using Haploview software, version 4.2 (http://www.broad.mit.edu/mpg/haploview/).

Linkage disequilibrium analyses were conducted by both FBAT and Haploview software. The pairwise linkage between two SNPs was estimated by the normalized disequilibrium coefficient (*D*’) and the squared correlation coefficient (r^2^). Then the global and individual haplotype tests of association were performed under multiallelic and biallelic mode in haplotype-based association test (HBAT) using FBAT software. The permutation test (*n* = 10,000) was used for multiple testing correction in HBAT. All *P*-values involved in HBAT were two-sided.

### In silico analyses of *ZNF804A* polymorphisms

Some online tools were used to predict the functions of SNPs in *ZNF804A*. HaploReg v4.1 (https://pubs.broadinstitute.org/mammals/haploreg/haploreg.php) was a tool for exploring the variants on haplotype blocks about their chromatin state and protein binding annotation, sequence conservation and effect on regulatory motifs and expression [37]. Genotype-Tissue Expression (GTEx) database (http://www.gtexportal.org/) provided the eQTL data to study relationship between genetic variation and gene expression in multiple human tissues. rVarBase (http://rv.psych.ac.cn/) and miRNASNP (http://bioinfo.life.hust.edu.cn/miRNASNP2/) were used to detected whether the associated SNPs were in the transcription factor (TF) binding regions, miRNA target regions, or miRNA seed regions [38, 39].

## Results

### Quality control

The MAF in CHB for rs1344706 and rs7603001 were greater than 0.05 (MAF = 0.476 and 0.204, respectively) in the Ensembl GRCh37 Release 93 database. In the present study, the MAF for these two SNPs were equal to 0.499 and 0.176, respectively. The call rates, determined by Sanger sequencing, were 97.7% for rs1344706 and 100% for rs7603001. The power to detect a true risk variant was 23%–63% for rs1344706 and 15%–40% for rs7603001 under the log-additive model. The genotypic distributions of these two SNPs in both unaffected parents and affected offspring did not derive from the HWE (*P* > 0.05, Table 1).

**Table 1:** Genotypic distributions of rs1344706 and rs7603001 in *ZNF804A* in 640 Han Chinese autism trios

SNP ID	Position	Genotype frequencies in children			*P* HWE^a^	Genotype frequencies in parents			*P* HWE^b^
rs1344706	chr2:184913701	CC	CA	AA		CC	CA	AA	
		165	316	144	0.758	326	600	324	0.157
rs7603001	chr2:184902089	GG	GA	AA		GG	GA	AA	
		444	174	22	0.336	865	380	35	0.380

^a^ Hardy–Weinberg equilibrium (HWE) *P* value for genotypic distributions in autistic children

^b^ HWE *P* value for genotypic distributions in parents

### SNP association and haplotype analyses

**Fig. 1:**
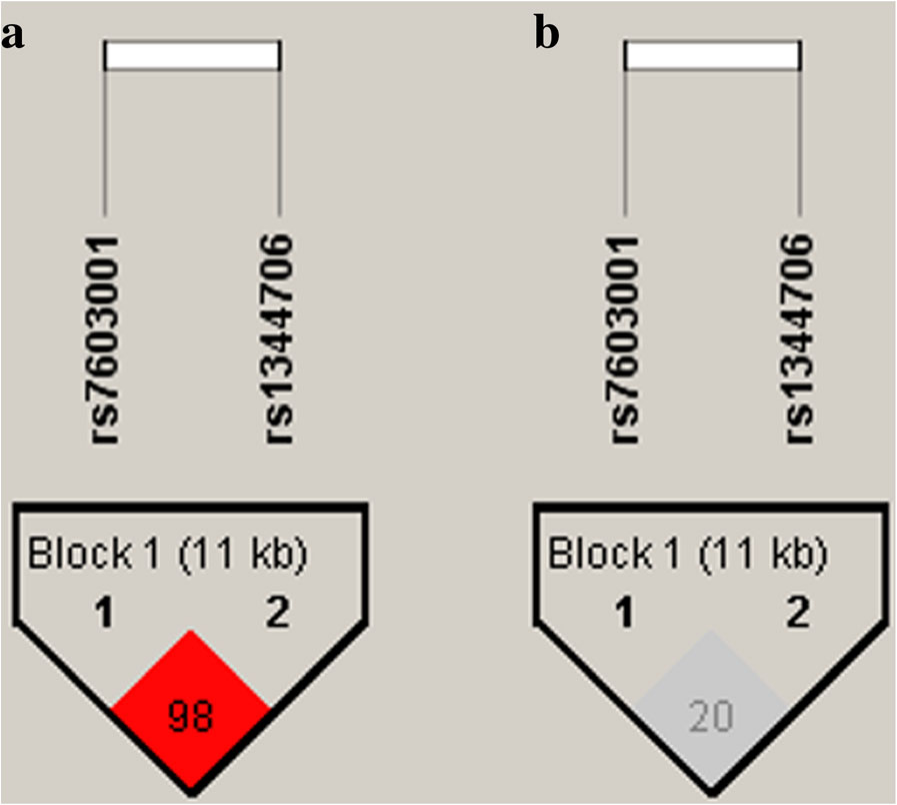
Linkage disequilibrium block constructed from rs7603001 and rs1344706 in *ZNF804A*. **a** Markers with linkage disequilibrium (LD) (*D*’ ≤ 1 and LOD ≥ 2) are shown in red. Values of *D*’ shown in the square represents pairwise LD relationship between the two polymorphisms. **b** Markers with LD (0 < r^2^ ≤ 1) are shown in grey with r^2^ value in the square. The LD plot was generated using the Halpoview program. The genotyping data was downloaded from Ensembl GCRh37 Release 93

The results from the single-SNP association analyses revealed that neither of the SNPs (rs1344706 and rs7603001) was significantly associated with autism under the additive or recessive inheritance model (Table 2). The value of *D*’ and r^2^ between rs1344706 and rs7603001 were 0.984 and 0.207, respectively (Fig. 1). Haplotype-based association analyses showed that no haplotypes were associated with autism (Table 3).

**Table 2:** Association analyses results of rs1344706 and rs7603001 in *ZNF804A* in 640 Han Chinese autism trios

SNP ID	Allele	Afreq	T:U^a^	Additive model				Recessive model				Dominant model			
				S-E(S)	Var(S)	Z	*P*	S-E(S)	Var(S)	Z	*P*	S-E(S)	Var(S)	Z	*P*
rs1344706	A	0.499	269:307	−19.00	144.00	−1.583	0.113	−11.75	62.19	−1.490	0.136	−7.25	63.19	−0.912	0.362
	C	0.501	307:269	19.00	144.00	1.583	0.113	7.25	63.19	0.912	0.362	11.75	62.19	1.490	0.136
rs7603001	A	0.176	173:201	−14.00	93.50	−1.448	0.148	−4.75	13.56	−1.290	0.197	−9.25	72.56	−1.086	0.278
	G	0.824	201:173	14.00	93.50	1.448	0.148	9.25	72.56	1.086	0.278	4.75	13.56	1.290	0.197

Abbreviations: *Afreq*, allele frequency; *S*, test statistics for the observed number of transmitted alleles; *E(S)*, expected value of S under the null hypothesis (i.e., no linkage and no association)

^a^ The ratio of transmission to untransmission (T:U) for each selected SNP was calculated by the Haploview version 4.2

**Table 3:** Haplotype-based association analyses between rs1344706 and rs7603001 in *ZNF804A* in 640 Han Chinese autism trios

Marker	Haplotype	Freq	Fam	S-E(S)	Var(S)	Z	*P*	Global *P*	*P*permutation^a^
rs7603001-rs1344706	G-C	0.414	212	15.92	72.89	1.864	0.062	0.100	0.089
	A-A	0.305	285	−14.08	83.76	−1.539	0.124		
	G-A	0.276	207	−0.42	65.90	−0.051	0.959		
	A-C	0.006	4	n/a	n/a	n/a	n/a		

Abbreviations: Freq, Estimation of haplotype frequencies; Fam, number of informative families; S, test statistics for the observed number of transmitted alleles; E(S), expected value of S under the null hypothesis (i.e., no linkage and no association); n/a: not applicable

^a^ Whole marker permutation test was performed using chisq sum *P* value (*n* = 10,000)

### Putative regulatory function and eQTL of selected SNPs

The function prediction using HaploReg showed that both rs1344706 and rs7603001 might alter the regulatory motifs (Additional file 1: Table S1). However, eQTL data from GTEx database demonstrated that both rs1344706 and rs7603001 were not associated with the expression of *ZNF804A* in human brain. Online databases rVarbase and miRNASNP revealed that neither of these two variants might be in TF binding regions, miRNA target regions, or miRNA seed regions.

## Discussion

This family-based association study was performed in 640 Han Chinese autism trios to investigate the relationships between two SNPs (rs1344706 and rs7603001) in *ZNF804A* and autism. Our results indicated that these two SNPs were not associated with autism in a Han Chinese population.

Our findings were inconsistent with those of a previous study. Using data of 841 autistic families from AGRE, Anitha et al. found that rs7603001 in *ZNF804A* was nominally associated with autism (*P* = 0.018), especially in the subgroup of autistic individuals with verbal deficit (*P* = 0.008). Another SNP, rs1344706, which was frequently reported in SCZ, showed no association with autism [15]. In addition, no association of rs1344706 (OR for A allele = 0.9805, *P* = 0.1597) or rs7603001 (OR for A allele = 0.9848, *P* = 0.2666) was found using genome-wide association data from the Integrative Psychiatric Research and the Psychiatric Genomics Consortium released in 2017 (iPSYCH-PGC GWAS 2017, available at: http://www.med.unc.edu/pgc/results-and-downloads), which included data of 18,381 autistic individuals and 27,969 controls [40].

Although this study detected no association between *ZNF804A* and autism, certain factors should be considered for further studies. First, autism is a complex heterogeneous disorder. Susceptibility genes might contribute to different subgroups of autism [41–45]. Concerning *ZNF804A*, a nominal association was detected between rs7603001 and autism, especially in autism individuals with verbal deficit. Hence, the relationship between candidate genes and specific phenotypes of autism should be further explored. Second, other SNPs or structural abnormalities such as copy number variations (CNVs) in *ZNF804A* might be involved in the etiology of autism. In the dataset of iPSYCH-PGC GWAS 2017, a few SNPs, including rs146362735, rs114385979, and rs77076543, were nominally associated with ASD (*P* < 0.01). However, these SNPs showed no polymorphism in the CHB population. Recently, one study found that rs10497655 in *ZNF804A* was significantly associated with ASD (OR = 1.20 (95%CI 1.05–1.37), *P* = 0.007851) in a Han Chinese cohort (854 cases and 926 controls) and the T risk allele homozygotes of rs10497655 could reduce *ZNF804A* expression [46]. On the other hand, Griswold et al. found duplications of CNVs in *ZNF804A* only in autistic individuals [47]. In another study, an excess of CNVs in *ZNF804A* was detected in 19,556 patients with neurodevelopmental disorders compared with 13,991 controls (*P* = 0.047) [48]. Therefore, the association between autism and other SNPs, and/or structural abnormalities in *ZNF804A* should be further investigated. Third, differences in ethnic genetic background might contribute to the result inconsistencies. For instance, r^2^ between rs1344706 and rs7603001 were 0.637, 0.232 and 0.353 in CEU (Utah residents with Northern and Western European ancestry from the CEPH collection), CHB and JPT (Japanese in Tokyo), respectively (Additional file 1: Figure S1). Besides, rs1344706 was reported to confer risk of SCZ in the European populations. However, a meta-analysis study found only nominal association between this variant and SCZ in the Asian population (13,452 cases, 17,826 healthy controls, and 680 families). No association was showed between rs1344706 and SCZ in samples from the Chinese population [49]. Association studies in other populations are required to assess the involvement of *ZNF804A* in autism. Fourth, the present sample size was limited. More autism patients and families of Han Chinese ancestry need to be involved in further researches to increase the statistical power and might help indicate new susceptibility variants.

## Conclusions

In summary, this study suggested that rs1344706 and rs7603001 in *ZNF804A* were not associated with autism in a Han Chinese population. Further research is needed to comprehensively explore the relationships between *ZNF804A* and autism.

## Additional file

Additional file 1: **Table S1.** Functional annotation of rs7603001 and rs1344706 in *ZNF804A* using HaploReg. **Figure S1.** Linkage disequilibrium block constructed from rs7603001 and rs1344706 in *ZNF804A* in different populations. (DOCX 132 kb)

## Abbreviations

ACG: Anterior cingulate gyrus
AGRE: Autism Genetic Resource Exchange
ASD: Autism spectrum disorder
CEU: Utah residents with Northern and Western European ancestry from the CEPH collection
CHB: Han Chinese in Beijing, China
CNVs: Copy number variations
FBAT: Family-based association test
GTEx: Genotype-Tissue Expression
HBAT: Haplotype-based association test
HWE: Hardy–Weinberg equilibrium
iPSYCH-PGC GWAS 2017: Integrative Psychiatric Research and the Psychiatric Genomics Consortium released in 2017
JPT: Japanese in Tokyo
MAF: Minor allele frequency
NCBI: National Center for Biotechnology Information
PCR: Polymerase chain reaction
SCZ: Schizophrenia
SNP: Single-nucleotide polymorphism
TF: Transcription factor
ToM: Theory of mind
*ZNF804A*: Zinc finger protein 804A

## References

[1] Lai MC, Lombardo MV, Baron-Cohen S (2014). Autism. Lancet..

[2] Volkmar FR, Lord C, Bailey A, Schultz RT, Klin A (2004). Autism and pervasive developmental disorders. J Child Psychol Psychiatry.

[3] Muhle R, Trentacoste SV, Rapin I (2004). The genetics of autism. Pediatrics..

[4] Rosenberg RE, Law JK, Yenokyan G, McGready J, Kaufmann WE, Law PA (2009). Characteristics and concordance of autism spectrum disorders among 277 twin pairs. Arch Pediatr Adolesc Med.

[5] Weiss LA, Arking DE, Daly MJ, Chakravarti A (2009). A genome-wide linkage and association scan reveals novel loci for autism. Nature.

[6] Geschwind DH (2011). Genetics of autism spectrum disorders. Trends Cogn Sci.

[7] Murdoch JD, State MW (2013). Recent developments in the genetics of autism spectrum disorders. Curr Opin Genet Dev.

[8] Schaaf CP, Zoghbi HY (2011). Solving the autism puzzle a few pieces at a time. Neuron..

[9] Hess JL, Glatt SJ (2014). How might ZNF804A variants influence risk for schizophrenia and bipolar disorder? A literature review, synthesis, and bioinformatic analysis. Am J Med Genet B Neuropsychiatr Genet.

[10] Brayer KJ, Segal DJ (2008). Keep your fingers off my DNA: protein-protein interactions mediated by C2H2 zinc finger domains. Cell Biochem Biophys.

[11] Girgenti MJ, LoTurco JJ, Maher BJ (2012). ZNF804a regulates expression of the schizophrenia-associated genes PRSS16, COMT, PDE4B, and DRD2. PLoS One.

[12] Deans PJM, Raval P, Sellers KJ, Gatford NJF, Halai S, Duarte RRR, Shum C, Warre-Cornish K, Kaplun VE, Cocks G (2017). Psychosis risk candidate ZNF804A localizes to synapses and regulates neurite formation and dendritic spine structure. Biol Psychiatry.

[13] Rao S, Yao Y, Ryan J, Jin C, Xu Y, Huang X, Guo J, Wen Y, Mao C, Meyre D (2017). Genetic association of rs1344706 in ZNF804A with bipolar disorder and schizophrenia susceptibility in Chinese populations. Sci Rep.

[14] Hill MJ, Jeffries AR, Dobson RJ, Price J, Bray NJ (2012). Knockdown of the psychosis susceptibility gene ZNF804A alters expression of genes involved in cell adhesion. Hum Mol Genet.

[15] Anitha A, Thanseem I, Nakamura K, Vasu MM, Yamada K, Ueki T, Iwayama Y, Toyota T, Tsuchiya KJ, Iwata Y (2014). Zinc finger protein 804A (ZNF804A) and verbal deficits in individuals with autism. J Psychiatry Neurosci.

[16] Apps MA, Rushworth MF, Chang SW (2016). The anterior cingulate gyrus and social cognition: tracking the motivation of others. Neuron.

[17] O'Donovan MC, Craddock N, Norton N, Williams H, Peirce T, Moskvina V, Nikolov I, Hamshere M, Carroll L, Georgieva L (2008). Identification of loci associated with schizophrenia by genome-wide association and follow-up. Nat Genet.

[18] Riley B, Thiselton D, Maher BS, Bigdeli T, Wormley B, McMichael GO, Fanous AH, Vladimirov V, O'Neill FA, Walsh D (2010). Replication of association between schizophrenia and ZNF804A in the Irish case-control study of schizophrenia sample. Mol Psychiatry.

[19] Li M, Luo XJ, Xiao X, Shi L, Liu XY, Yin LD, Diao HB, Su B (2011). Allelic differences between Han Chinese and Europeans for functional variants in ZNF804A and their association with schizophrenia. Am J Psychiatry.

[20] Williams HJ, Norton N, Dwyer S, Moskvina V, Nikolov I, Carroll L, Georgieva L, Williams NM, Morris DW, Quinn EM (2011). Fine mapping of ZNF804A and genome-wide significant evidence for its involvement in schizophrenia and bipolar disorder. Mol Psychiatry.

[21] Ou J, Li M, Xiao X (2017). The schizophrenia susceptibility gene ZNF804A confers risk of major mood disorders. World J Biol Psychiatry.

[22] Cross-Disorder Group of the Psychiatric Genomics Consortium (2013). Identification of risk loci with shared effects on five major psychiatric disorders: a genome-wide analysis. Lancet.

[23] Walter H, Schnell K, Erk S, Arnold C, Kirsch P, Esslinger C, Mier D, Schmitgen MM, Rietschel M, Witt SH (2011). Effects of a genome-wide supported psychosis risk variant on neural activation during a theory-of-mind task. Mol Psychiatry.

[24] Enticott PG, Kennedy HA, Rinehart NJ, Tonge BJ, Bradshaw JL, Taffe JR, Daskalakis ZJ, Fitzgerald PB (2012). Mirror neuron activity associated with social impairments but not age in autism spectrum disorder. Biol Psychiatry.

[25] Dvash J, Shamay-Tsoory SG (2014). Theory of mind and empathy as multidimensional constructs neurological foundations. Top Lang Disord.

[26] Wellman HM, Cross D, Watson J (2001). Meta-analysis of theory-of-mind development: the truth about false belief. Child Dev.

[27] Yirmiya N, Erel O, Shaked M, Solomonica-Levi D (1998). Meta-analyses comparing theory of mind abilities of individuals with autism, individuals with mental retardation, and normally developing individuals. Psychol Bull.

[28] Cheng W, Rolls ET, Gu H, Zhang J, Feng J (2015). Autism: reduced connectivity between cortical areas involved in face expression, theory of mind, and the sense of self. Brain..

[29] Hoogenhout M, Malcolm-Smith S (2017). Theory of mind predicts severity level in autism. Autism..

[30] Krug DA, Arick J, Almond P (1980). Behavior checklist for identifying severely handicapped individuals with high levels of autistic behavior. J Child Psychol Psychiatry.

[31] Schopler E, Reichler RJ, DeVellis RF, Daly K (1980). Toward objective classification of childhood autism: childhood autism rating scale (CARS). J Autism Dev Disord.

[32] Zerbino DR, Achuthan P, Akanni W, Amode MR, Barrell D, Bhai J, Billis K, Cummins C, Gall A, Girón CG (2018). Ensembl 2018. Nucleic Acids Res.

[33] Gauderman A. QUANTO 1.1: a computer program for power and sample size calculations for genetic-epidemiology studies. http://hydrauscedu/gxe. 2016.

[34] Buxbaum JD, Baron-Cohen S, Devlin B (2010). Genetics in psychiatry: common variant association studies. Mol Autism.

[35] Family-based association test (FBAT). In: Encyclopedia of Genetics, Genomics, Proteomics and Informatics*.* Edn. Dordrecht: Springer Netherlands; 2008. p. 671–1.

[36] Ranstam J (2016). Multiple P-values and Bonferroni correction. Osteoarthr Cartil.

[37] Ramasamy A, Trabzuni D, Guelfi S, Varghese V, Smith C, Walker R, De T, Consortium UKBE (2014). North American brain expression C, coin L et al. genetic variability in the regulation of gene expression in ten regions of the human brain. Nat Neurosci.

[38] Guo L, Du Y, Qu S, Wang J (2016). rVarBase: an updated database for regulatory features of human variants. Nucleic Acids Res.

[39] Gong J, Tong Y, Zhang HM, AYJBB G. miRNASNP: a database of miRNA related SNPs and their effects on miRNA function. 2012;13(Suppl 18):A2–2.

[40] Grove J, Ripke S, Als TD, Mattheisen M, Walters R, Won H, Pallesen J, Agerbo E, Andreassen OA, Anney R, et al. Common risk variants identified in autism spectrum disorder. BioRxiv. 2017:224774.

[41] Bartlett CW, Flax JF, Logue MW, Smith BJ, Vieland VJ, Tallal P, Brzustowicz LM (2004). Examination of potential overlap in autism and language loci on chromosomes 2, 7, and 13 in two independent samples ascertained for specific language impairment. Hum Hered.

[42] Talebizadeh Z, Arking DE, Hu VW (2013). A novel stratification method in linkage studies to address inter- and intra-family heterogeneity in autism. PLoS One.

[43] Vernes SC, Newbury DF, Abrahams BS, Winchester L, Nicod J, Groszer M, Alarcon M, Oliver PL, Davies KE, Geschwind DH (2008). A functional genetic link between distinct developmental language disorders. N Engl J Med.

[44] Whitehouse AJ, Bishop DV, Ang QW, Pennell CE, Fisher SE (2011). CNTNAP2 variants affect early language development in the general population. Genes Brain Behav.

[45] Ross LA, Del Bene VA, Molholm S, Jae Woo Y, Andrade GN, Abrahams BS, Foxe JJ (2017). Common variation in the autism risk gene CNTNAP2, brain structural connectivity and multisensory speech integration. Brain Lang.

[46] Zhang L, Qin Y, Gong X, Peng R, Cai C, Zheng Y, Du Y, Wang H (2019). A promoter variant in ZNF804A decreasing its expression increases the risk of autism spectrum disorder in the Han Chinese population. Transl Psychiatry.

[47] Griswold AJ, Ma D, Cukier HN, Nations LD, Schmidt MA, Chung RH, Jaworski JM, Salyakina D, Konidari I, Whitehead PL (2012). Evaluation of copy number variations reveals novel candidate genes in autism spectrum disorder-associated pathways. Hum Mol Genet.

[48] Talkowski ME, Rosenfeld JA, Blumenthal I, Pillalamarri V, Chiang C, Heilbut A, Ernst C, Hanscom C, Rossin E, Lindgren AM (2012). Sequencing chromosomal abnormalities reveals neurodevelopmental loci that confer risk across diagnostic boundaries. Cell..

[49] Huang L, Ohi K, Chang H, Yu H, Wu L, Yue W, Zhang D, Gao L (2016). Li M. a comprehensive meta-analysis of ZNF804A SNPs in the risk of schizophrenia among Asian populations. Am J Med Genet B Neuropsychiatr Genet.

