# Supplementary material for: Family-based association study of ZNF804A polymorphisms and autism in a Han Chinese population
Source: BMC Psychiatry. 2019 May 23;19:159. doi: 10.1186/s12888-019-2144-1 (PMC6533675; doi:10.1186/s12888-019-2144-1)
Supplement: Supplementary file 1 — Table S1. Functional annotation of rs7603001 and rs1344706 in ZNF804A using HaploReg. Figure S1. Linkage disequilibrium block constructed from rs7603001 and rs1344706 in ZNF804A in different populations. (DOCX 132 kb) [file 12888_2019_2144_MOESM1_ESM.docx]

**Additional file 1**

**Table S1.** Functional annotation of rs7603001 and rs1344706 in *ZNF804A* using HaploReg.

**Figure S1.** Linkage disequilibrium block constructed from rs7603001 and rs1344706 in *ZNF804A* in different populations.

**Table S1.** Functional annotation of rs7603001 and rs1344706 in *ZNF804A* using HaploReg.

| SNP | Position | Function annotation | Promoter histone marks | Enhancer histone marks | DNAse | Motifs changed |  |
| --- | --- | --- | --- | --- | --- | --- | --- |
| rs1344706 | chr2:184913701 | intronic | n/a | n/a | n/a | Homez | |
| rs7603001 | chr2:184902089 | intronic | n/a | n/a | n/a | Nkx2, Nkx3, STAT | |

Abbreviation: n/a, not applicable.


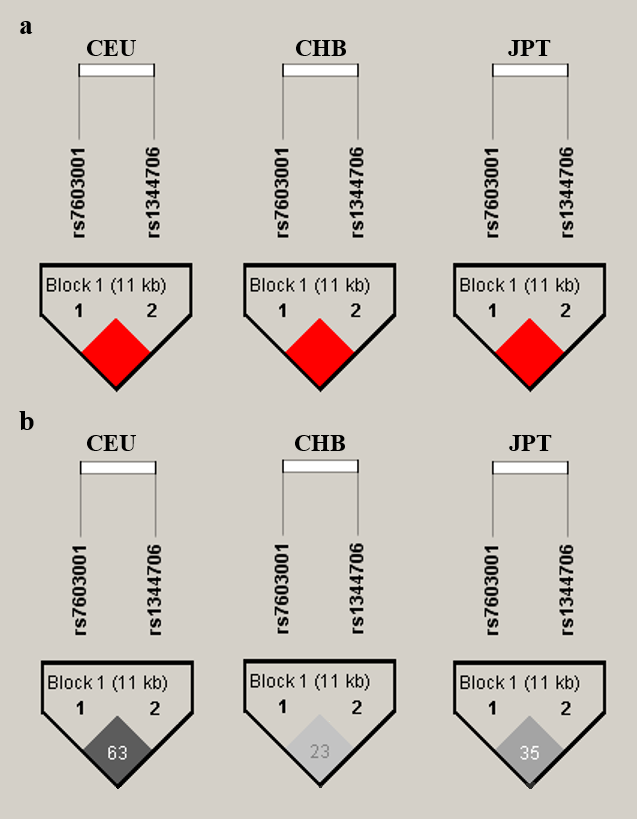
 **Figure S1.** Linkage disequilibrium block constructed from rs7603001 and rs1344706 in *ZNF804A* in different populations. **a** Markers with linkage disequilibrium (LD) (*D*' ≤ 1 and LOD ≥ 2) are shown in red. Values of *D*' shown in each square represents pairwise LD relationship between the two polymorphisms. *D*' value of 1.0 are not shown (the square is blank). **b** Markers with LD (0 < r^2^ ≤ 1) are shown in black through grey (color intensity decreased with decreasing r^2^ value). r^2^ value are shown in each square. The LD plot was generated using the Halpoview program. The genotyping data was downloaded from Ensembl GCRh37 Release 93. CEU, Utah residents with Northern and Western European ancestry from the CEPH collection; CHB, Han Chinese in Beijing, China; JPT, Japanese in Tokyo.
